# Multifaceted Attack Networks of Artemisinin in Reversing Chemoresistance in Colorectal Cancer

**DOI:** 10.3390/molecules31020244

**Published:** 2026-01-11

**Authors:** Mingfei Liu, Yueling Yan, Shirong Li, Rongrong Wang, Kewu Zeng, Jingchun Yao

**Affiliations:** 1 School of Chinese Materia Medica, Tianjin University of Traditional Chinese Medicine, Tianjin 301617, China; lmf11210216@163.com (M.L.); y3178002270@gmail.com (Y.Y.); 2 State Key Laboratory of Integration and Innovation of Classic Formula and Modern Chinese Medicine, Lunan Pharmaceutical Group Co., Ltd., Linyi 276005, China; shirongli993@163.com; 3 Key Laboratory of Marine Drugs, School of Medicine and Pharmacy, Ministry of Education, Ocean University of China, Qingdao 266071, China; wrr2996@stu.ouc.edu.cn

**Keywords:** artemisinin, colorectal cancer, chemotherapy resistance, cancer stem cells, ferroptosis, tumor immune microenvironment

## Abstract

Chemotherapy resistance in colorectal cancer (CRC) represents a critical clinical challenge leading to treatment failure and poor patient prognosis. Artemisinin is a natural product isolated from Artemisia annua, and its clinically relevant derivatives include dihydroartemisinin (DHA) and artesunate. Beyond their established antimalarial efficacy, both artemisinin and its derivatives—collectively referred to as artemisinin-derived compounds (ADs)—have been increasingly recognized for their unique potential to reverse multidrug resistance in cancer. Unlike previous reviews focusing on isolated mechanisms, this review systematically constructs a multidimensional, synergistic attack network centered on ADs to elucidate their integrated actions against chemotherapy-resistant CRC. Mechanistically, ADs suppress cancer stem cell (CSC)-associated resistance phenotypes while concurrently reshaping the tumor immune microenvironment, highlighting a functional coupling between stemness inhibition and immune remodeling. In parallel, this review presents apoptosis reactivation and ferroptosis induction as complementary, dual-track cell death strategies that collectively circumvent apoptosis resistance. Moreover, ADs exert “one-strike–multiple-effects” through coordinated regulation of pro-survival signaling networks and immune-related pathways, including the induction of immunogenic cell death (ICD) and the modulation of immunosuppressive macrophage subsets. Beyond mechanistic insights, this review integrates emerging translational considerations, including clinical pharmacokinetics, safety and tolerability, formulation and delivery strategies, and rational combination therapy paradigms in CRC. Collectively, these findings position ADs as multi-dimensional modulators rather than a single-agent cytotoxic, providing a coherent mechanistic and translational rationale for their further development in chemotherapy-resistant CRC.

## 1. Introduction

Colorectal cancer (CRC) ranks among the most lethal malignant tumors globally. In recent years, driven by lifestyle changes and accelerated population aging, its incidence has continued to rise, emerging as a major public health concern [1,2]. In the clinical treatment of CRC, chemotherapy remains the primary therapeutic modality alongside surgery [3,4]. However, the emergence of chemotherapy resistance has become a major bottleneck limiting treatment efficacy [5]. The development of chemoresistance in CRC involves the synergistic interplay of multiple mechanisms, including the maintenance of cancer stem cells (CSCs) [6], evasion of apoptosis [7], remodeling of the tumor microenvironment [8], and dysregulation of survival-related signaling pathways. Together, these processes constitute a highly adaptive and integrated cellular defense system.

**Figure 1:**
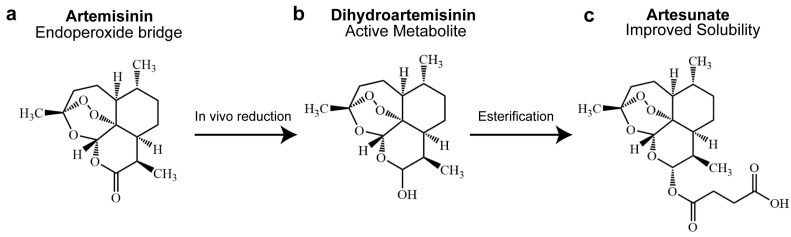
Chemical Structures of Artemisinin, Dihydroartemisinin, and Artesunate, Illustrating Their Metabolic and Semi-synthetic Relationships. (**a**) The Structure of Artemisinin, (**b**) The Structure of Dihydroartemisinin, (**c**) The Structure of Artesunate.

Artemisinin is a sesquiterpene lactone compound extracted from the plant Artemisia annua [9]. Structurally, artemisinin is characterized by a unique endoperoxide bridge, which is essential for its pharmacological activity [10]. As illustrated in Figure 1, artemisinin is rapidly metabolized in vivo to dihydroartemisinin (DHA), the principal active metabolite responsible for most biological effects [11]. DHA further serves as a key intermediate for semi-synthetic derivatives, such as artesunate, which is generated through esterification to improve aqueous solubility and pharmacokinetic properties [12]. Due to these favorable characteristics, artemisinin-derived compounds (ADs), encompassing artemisinin and its clinically relevant derivatives such as DHA and artesunate, represent the most effective antimalarial agents and form the backbone of artemisinin-based combination therapies [13,14]. In addition to its use in treating malaria, ADs have also been reported to exhibit antitumor activity in various cancer models [15,16,17,18,19]. From a structure–activity perspective, the conserved endoperoxide moiety enables ADs to be selectively activated in iron-rich tumor cells, triggering the generation of excessive reactive oxygen species (ROS) and oxidative stress. This mechanism is particularly relevant in CRC, where dysregulated iron metabolism and redox homeostasis render tumor cells highly susceptible to oxidative damage. Recent studies have demonstrated that DHA and artesunate exert pronounced cytotoxic effects in CRC models by inducing apoptosis as well as ferroptosis, a distinct form of iron-dependent non-apoptotic cell death [19,20,21].

Importantly, ADs do not merely act as direct cytotoxic agents but also interfere with multiple resistance-associated mechanisms in CRC. For instance, ADs weaken CSC properties by inhibiting the AKT/mTOR signaling axis [20], restore apoptosis sensitivity through modulation of the Bcl-2/Bax balance [22,23], and alleviate tumor-induced immunosuppression by blocking STAT3 signaling [23,24]. These multifaceted effects suggest that ADs possess substantial potential to reverse chemotherapy resistance in CRC. However, despite extensive mechanistic studies, existing research remains largely fragmented, often focusing on isolated pathways or single cell death modalities. Consequently, a unified conceptual framework integrating CSC eradication, apoptosis/ferroptosis reactivation, pro-survival signaling suppression, and tumor microenvironment remodeling is still lacking, which hampers a comprehensive understanding of the multi-target synergistic actions of ADs and limits the rational design of combination therapy strategies.

Therefore, this review moves beyond traditional single-mechanism perspectives by proposing a unified “multidimensional attack network” through which ADs reverse chemotherapy resistance in CRC. This framework integrates four interconnected dimensions—CSC eradication, reactivation of apoptosis and ferroptosis, suppression of pro-survival signaling, and remodeling of the tumor immune microenvironment while extending these mechanistic insights to key translational considerations, including pharmacokinetics, safety, formulation strategies, and rational combination therapy. Collectively, this integrated mechanistic–translational framework provides a coherent foundation for the rational development and clinical translation of AD-based resistance-reversal strategies in CRC.

## 2. Mechanisms by Which ADs Overcome Chemotherapy Resistance in CRC

### 2.1. Targeting the Root Cause: Eliminating CSCs to Prevent Drug Resistance and Recurrence

CSCs are a subpopulation of cells within tumor tissue that possess stem cell characteristics, including the potential for self-renewal, multidirectional differentiation, and unlimited proliferation [25]. Characterized by the overexpression of stem cell markers, metastasis-associated proteins, drug transporters, and molecules involved in DNA repair mechanisms; these properties confer a pivotal role to CSCs in tumorigenesis, progression, invasion and metastasis, recurrence, and the development of drug resistance [6,26,27,28]. CSCs could initiate malignant evolution in cancer cells and replenish tumor cell populations, thereby driving tumor progression, leading to resistance to chemotherapy treatment, and promoting recurrence [29,30]. Drug resistance involves multiple mechanisms, including maintenance of cellular quiescence, enhanced DNA repair capacity, evasion of apoptosis, epithelial–mesenchymal transition (EMT), reduced ROS levels, and upregulation of aldehyde dehydrogenase (ALDH) activity [31,32,33,34,35].

ADs can exert anti-CRC effects and overcome chemotherapy resistance by targeting CSCs. Specifically, DHA effectively downregulates the expression of stem cell surface markers such as CD133 and CD44, as well as core stemness transcription factors including Nanog, c-Myc, and OCT4 by inhibiting the AKT/mTOR signaling axis. This significantly impairs the self-renewal and tumor spheroid formation capabilities of CSCs [20]. Simultaneously, DHA induces ROS accumulation within CSCs, leading to mitochondrial membrane potential loss and autophagy activation, thereby undermining their survival foundation at the metabolic and cellular homeostasis levels [36]. On the other hand, artesunate reverses the EMT process by inhibiting the overactivated Wnt/β-catenin pathway, thereby preventing the nuclear translocation of β-catenin and promoting the differentiation of CSCs into non-stem cells that are more susceptible to elimination [37,38,39].

Crucially, the combination strategy of ADs with conventional chemotherapy fundamentally reverses drug resistance and prevents recurrence by synergistically targeting CSCs and their microenvironment. For example, the combination of DHA and oxaliplatin not only suppresses CSC properties through the aforementioned AKT/mTOR pathway but also induces immunogenic cell death (ICD) by activating the PERK/eIF2α pathway. This reshapes the tumor immune microenvironment, thereby synergistically eliminating drug-resistant CSCs [20,40]. Similarly, the combination of artesunate and 5-fluorouracil (5-FU) renders CSCs sensitive to 5-FU by inhibiting the Wnt pathway, while simultaneously mitigating 5-FU-induced intestinal damage and cellular senescence through mTOR pathway suppression. This creates conditions for sustained, effective chemotherapy to eliminate CSCs [37,38,39,41]. These combined regimens confirm that ADs serve as potent CSCs sensitizers, complementing conventional chemotherapy through multi-mechanistic synergistic elimination of CSCs. This approach offers a highly promising new paradigm for overcoming drug resistance and recurrence in CRC.

Eliminating CSCs not only directly eradicates the root cause of drug resistance but also creates favorable conditions for subsequent treatments. The reduction in CSCs differentiated or killed by DHA and artesunate directly diminishes tumor heterogeneity and regenerative capacity. This makes the remaining population of tumor cells—which are relatively sensitive to chemotherapy—more susceptible to complete eradication by conventional chemotherapeutic agents. Simultaneously, the ablation of CSCs alters the secretory profile of tumor cells, helping to reverse their apoptotic resistance and laying the groundwork for ultimately reshaping the tumor immune microenvironment.

### 2.2. Reversing Apoptosis Resistance and Activating Novel Death Pathways

Conventional chemotherapeutic agents (such as 5-FU and oxaliplatin) primarily exert their antitumor effects by activating the intrinsic apoptotic pathway within cells [42]. However, CRC cells can construct a robust “anti-apoptotic defense” through a series of adaptive alterations [43,44]. These alterations include p53 inactivation, upregulation of anti-apoptotic proteins (such as Bcl-2 family members, XIAP, and survivin), downregulation of pro-apoptotic proteins (like Bax and Bak), and inhibition of caspase activity [45]. This directly impedes the occurrence of programmed cell death in cancer cells, thereby conferring resistance to chemotherapeutic agents [46]. In chemotherapy-resistant CRC cells, the expression of the anti-apoptotic protein Bcl-2 is significantly upregulated, while that of the pro-apoptotic protein Bax is significantly downregulated, confirming the presence of apoptosis suppression in drug-resistant CRC [45]; Concurrently, survivin, as an apoptosis-inhibiting protein, exhibits high expression closely associated with radiation resistance and apoptosis resistance in CRC cells [47,48]. These mechanisms cause chemotherapy drugs to disrupt or dilute the death signals triggering apoptosis during signal transduction, ultimately leading to treatment failure. Therefore, the ability to bypass or overcome this dysfunctional apoptotic pathway directly determines the success of overcoming drug resistance and provides the theoretical basis for developing new strategies targeting apoptosis resistance.

#### 2.2.1. Reactivate and Enhance the Classic Apoptosis Pathway

ADs can precisely intervene and activate apoptotic signaling networks at multiple nodes, thereby dismantling the defense mechanisms of drug-resistant cells. DHA has been demonstrated to effectively reduce the expression of downstream anti-apoptotic proteins such as survivin by inhibiting the JAK2/STAT3 signaling pathway, thereby releasing its inhibition on caspase enzymes and reactivating the apoptotic execution program [23]. Simultaneously, DHA directly induces mitochondrial-dependent apoptosis, manifested by promoting the expression of pro-apoptotic protein Bax, reducing anti-apoptotic protein Bcl-2 levels, inducing mitochondrial membrane potential loss and cytochrome c release, and ultimately activating caspase-9 and caspase-3. This series of events collectively constitutes the bypassed mitochondrial apoptosis pathway [22,23,49]. Additionally, DHA can induce endoplasmic reticulum stress by inhibiting the activity of the sarcoplasmic reticulum calcium pump, leading to a significant upregulation of the key transcription factor CHOP [22]. CHOP can further suppress Bcl-2 expression and activate pro-apoptotic factors such as Bim, thereby tightly coupling endoplasmic reticulum stress signaling with the mitochondrial apoptosis pathway and opening another effective pathway for inducing apoptosis [50]. These mechanisms ensure that even in the presence of partial apoptosis defects, ADs can effectively restart the self-destruction program of cancer cells through multi-target synergistic action.

#### 2.2.2. Activation of a Novel Non-Apoptotic Death Pathway-Ferroptosis

Beyond repairing the classical apoptosis pathway, ADs’ most compelling advantage lies in their ability to initiate a novel form of cell death independent of caspase activation-ferroptosis—which provides the ultimate weapon for overcoming apoptosis resistance. Ferroptosis is a distinct form of cell death driven by iron-dependent lipid peroxidation, exhibiting significant morphological and mechanistic differences from apoptosis, autophagy, and other processes [51,52]. Typical morphological features include disruption of cell membrane integrity, organelle swelling, chromatin condensation, and changes such as increased mitochondrial membrane density and reduced or absent cristae [51]. Research indicates that DHA can disrupt glycerophospholipid metabolic reprogramming by directly targeting and inhibiting the LOXL2 protein. This action compromises cell membrane integrity, triggers severe lipid peroxidation, and ultimately induces ferroptosis in cancer cells [53]. Crucially, the iron-dependent intracellular breakdown of DHA generates ROS. These ROS not only directly fuel lipid peroxidation but also initiate a self-amplifying loop by depleting glutathione and inactivating the lipid repair enzyme GPX4. This cripples the cell’s antioxidant defenses, leading to uncontrolled lipid peroxidation and further iron release, thereby creating a vicious cycle that drives ferroptosis [36,49,50,54]. Since ferroptosis operates through biochemical mechanisms entirely distinct from the classical apoptosis pathway, cancer cells cannot evade its lethal effects regardless of how they establish resistance to apoptosis—whether through p53 inactivation, Bcl-2 overexpression, or caspase inhibition.

Based on the aforementioned mechanism, the combination strategy of ADs with conventional chemotherapeutic agents demonstrates remarkable synergistic effects. Its core lies in simultaneously targeting apoptosis and ferroptosis, thereby achieving a multidimensional assault on drug-resistant cells. For example, the combination of DHA and oxaliplatin not only downregulates anti-apoptotic proteins by inhibiting AKT/mTOR and STAT3 signaling to restore sensitivity to oxaliplatin-induced apoptosis, but also achieves complementary killing by inducing ferroptosis to eliminate cell subpopulations that are insensitive to oxaliplatin-induced apoptosis [20,23,53,54]. Similarly, DHA has been shown to sensitize CRC cells to 5-FU predominantly by amplifying oxidative stress and disrupting redox homeostasis. Experimental studies demonstrate that DHA markedly elevates intracellular ROS, suppresses pro-survival signaling such as AKT/mTOR, and thereby enhances 5-FU-induced cytotoxicity in drug-resistant CRC models [55,56]. Under sustained chemotherapy stress, this ROS overload may further promote lipid peroxidation-associated vulnerability, enabling ferroptosis-related cell death to act as a complementary, non-apoptotic mechanism, ultimately contributing to the reversal of chemotherapy resistance driven by apoptosis evasion.

Based on the aforementioned mechanism, the successful reactivation of the cell death program by ADs produces profound effects that extend beyond direct cytotoxic effects. On the one hand, the enhanced apoptotic signaling driven by endoplasmic reticulum stress and death receptor pathways [22,50] complements the mechanisms suppressing CSC survival described in Section 2.1, ensuring comprehensive elimination from stem cells to differentiated cells. On the other hand, novel death pathways such as AD-induced ferroptosis can release large quantities of damage-associated molecular patterns (DAMPs) [40]. This process serves as a critical bridge, directly linking the cytotoxic effects to ICD and anti-tumor immune activation—discussed in Section 2.4—thereby converting internal death signals into systemic immune attacks against tumors.

### 2.3. Inhibit Pro-Survival Signaling Networks

The development of CRC chemotherapy resistance is a complex process involving multiple factors, stemming not only from dysregulation of the apoptosis pathway but also closely associated with various abnormally activated intracellular pro-survival signaling networks. In tumor cells, signaling pathways such as PI3K/AKT/mTOR [57], JAK/STAT3 [58], and TGF-β/Smad [59] form a robust defense system. These pathways are further activated under chemotherapy stress and promote the development of drug resistance through multiple mechanisms. For example, the phosphorylation and activation of STAT3 can upregulate multiple anti-apoptotic proteins [60], inhibit caspase activity, and thereby attenuate drug-induced apoptosis [23]. On the other hand, enhanced DNA damage repair capacity is one of the key mechanisms leading to resistance to radiotherapy and chemotherapy. Research indicates that genotoxic drugs such as 5-FU can feedback-activate pro-survival signaling pathways like ERK and STAT, thereby upregulating the expression of anti-apoptotic factors such as survival proteins, ultimately diminishing the cytotoxic effects of the drugs [61,62]. Therefore, inhibitors targeting these aberrant pathways are considered a key strategy for reversing CRC resistance, and their combination with conventional chemotherapy shows promising therapeutic prospects.

In antitumor applications, ADs function as multi-targeted “signaling network inhibitors”, effectively dismantling the internal defense systems of tumors. First, in cells resistant to oxaliplatin, DHA reverses this resistance by upregulating the deacetylase SIRT3, thereby inhibiting activation of the PI3K/AKT pathway. This mechanism directly reverses cancer cells’ resistance to oxaliplatin and effectively induces apoptosis [63]. Meanwhile, DHA directly inhibits phosphorylation of the JAK2/STAT3 signaling pathway, thereby downregulating a panel of STAT3-regulated anti-apoptotic proteins, including survivin as a representative target, which collectively undermines the survival advantage of cancer cells and restores their sensitivity to chemotherapeutic agents [23]. Notably, ADs exert a dual inhibitory effect on cellular proliferation and survival signals by targeting both metabolic and inflammatory pathways—specifically by suppressing fatty acid synthesis and the NF-κB pathway—thereby further enhancing the pro-apoptotic effects of ADs [9].

Crucially, the combination of ADs with conventional chemotherapy drugs can produce synergistic killing effects and reverse drug resistance by vertically inhibiting multiple complementary survival pathways. For example, the combination of DHA and oxaliplatin not only downregulates anti-apoptotic proteins and restores sensitivity to oxaliplatin-induced apoptosis by inhibiting AKT/mTOR and STAT3 signaling through the aforementioned mechanisms, but also synergistically enhances its antitumor activity by modulating multiple PRDX2-reactive oxygen species-mediated signaling pathways [20,64]. Similarly, the combination of artesunate and 5-FU further enhances 5-FU-induced cell cycle arrest and apoptosis through synergistic inhibition of the PI3K/AKT signaling pathway, significantly increasing cancer cells’ sensitivity to 5-FU [56]. These combined strategies confirm that ADs function as broad-spectrum “signaling pathway sensitizers”. By systematically suppressing cancer cells’ pro-survival networks, they restore and amplify the cytotoxic efficacy of conventional chemotherapeutic agents, offering a highly promising combination approach to overcoming CRC resistance mediated by multiple pro-survival signals.

Inhibition of key pro-survival signaling pathways such as PI3K/AKT/mTOR and JAK2/STAT3 is significant for systematically dismantling the defense mechanisms of cancer cells, exhibiting extensive cross-talk with mechanisms discussed in other sections. For instance, inhibiting AKT/mTOR signaling not only directly counters chemotherapy resistance but also concurrently weakens the stemness maintenance capacity of CSCs, as discussed in Section 2.1. Similarly, suppressing STAT3 downregulates anti-apoptotic proteins (such as Survivin) (in Section 2.2) while reducing the expression of multiple immunosuppressive factors (in Section 2.4). Thus, by targeting these central signaling hubs within the network, ADs generate synergistic “one-stone-kills-multiple-birds” effects—simultaneously enhancing apoptosis sensitivity, diminishing stem cell properties, and improving the immune microenvironment.

### 2.4. Rewiring the Tumor Microenvironment

The development of CRC chemotherapy resistance depends not only on the intrinsic characteristics of cancer cells but is also profoundly influenced by the suppressive tumor microenvironment surrounding them [65,66]. The tumor microenvironment comprises multiple cellular components (such as cancer-associated fibroblasts, tumor-associated macrophages, tumor-infiltrating lymphocytes, etc.) and non-cellular components (including extracellular matrix, cytokines, metabolic products, etc.). These components not only provide support for tumor cells but also regulate tumor cell proliferation, invasion, metastasis, and sensitivity to treatment through complex signaling pathways [67]. Cytokines in the tumor microenvironment, such as TGFβ and IL-6, can enhance drug resistance by promoting immune suppression and inducing EMT [68,69]. Additionally, metabolic reprogramming serves as a key mechanism through which the tumor microenvironment influences chemotherapy resistance. Tumor cells adapt to hypoxic and nutrient-deprived microenvironments by altering metabolic pathways such as glucose and fructose metabolism, thereby enhancing their survival capacity and drug resistance [70,71]. Additionally, immune suppression within the tumor microenvironment represents another key mechanism contributing to chemotherapy resistance. Research indicates that TGF-β signaling in tumor cells and their microenvironment enhances tumor immune evasion by promoting PD-L1 expression, recruiting immune-suppressive macrophages, and regulating T cells (Tregs) [72]. Concurrently blocking TGF-β and CSF1/CSF1R signaling pathways significantly enhances CD8^+^ T cell infiltration and activation during chemotherapy, reduces the proportion of Tregs, improves the tumor immune microenvironment, and boosts the efficacy of chemotherapy. Additionally, accumulating evidence suggests that modulation of specific miRNAs represents an important regulatory layer in tumor immune microenvironment remodeling, as these miRNAs can enhance the function of dendritic cells, M1 macrophages, and helper T cells while suppressing immunosuppressive Tregs and M2 macrophages, thereby improving immunotherapy responsiveness [73,74]. Although direct evidence linking ADs to the regulation of these immune-related miRNAs in CRC is currently limited, ADs may indirectly influence miRNA expression through their well-established effects on oxidative stress, STAT3 inhibition, and TGF-β signaling suppression. Given that these signaling pathways are known upstream regulators of multiple immunomodulatory miRNAs, future studies investigating ADs-mediated miRNA remodeling may provide novel mechanistic insights into their immunosensitizing effects and further expand their therapeutic potential in combination with immunotherapy.

ADs can serve as effective “tumor microenvironment modulators”, reversing tumor-induced immune suppression through multiple pathways. Artesunate has been demonstrated to directly act on CRC cells, significantly downregulating their TGF-β and IL-10 secretion levels. In the Colon26 and RKO colorectal cancer cell models, artesunate pretreatment substantially reduced TGF-β1 and IL-10 concentrations, effectively restoring natural killer (NK) cell cytotoxicity and lymphocyte proliferation capacity. It also promoted upregulation of the T cell activation key molecule CD3ζ chain, thereby partially reversing the immune suppression directly induced by tumor cells [75]. This directly interferes with immune evasion and chemotherapy resistance mediated by the TGF-β/Smad signaling pathway.

In modulating the tumor immune microenvironment, ADs demonstrate dual advantages: not only can they effectively alleviate immunosuppression, but they can also actively stimulate antitumor immune responses. This characteristic is particularly pronounced when combined with chemotherapy drugs. On the one hand, DHA exhibits potent ICD-inducing capacity when combined with cisplatin. Mechanistic studies indicate that this process activates the PERK/eIF2α endoplasmic reticulum stress signaling pathway, prompting apoptotic cells to efficiently release damage-associated molecular patterns such as calreticulin. This sequence of events effectively enhances dendritic cell phagocytosis and presentation of tumor antigens, thereby initiating and amplifying tumor-specific cytotoxic T cell responses [40]. Additionally, the platinum complex formed by coupling artesunate with oxaliplatin not only retains the cytotoxic properties of the parent drug but also acquires a novel function: targeted inhibition of the macrophage immunosuppressive receptor TREM2 [22]. In MC38 cells, this complex effectively reduces CD206+ M2 macrophages and CX3CR1+ immunosuppressive monocytes within tumors while promoting the infiltration and expansion of immunostimulatory dendritic cells, cytotoxic T cells, and natural killer cells. This transforms “immunologically cold” tumors into “immunologically hot” tumors, achieving profound remodeling of the tumor microenvironment and long-term immune control [22]. These studies collectively confirm that ADs function as potent “immune sensitizers” by directly reversing immunosuppression, actively inducing ICD, and precisely targeting immunosuppressive cells. Working synergistically with conventional therapies, they systematically reshape the tumor immune microenvironment. This offers a highly promising new paradigm for combination therapy to overcome CRC resistance mediated by immune evasion and microenvironmental protection.

In summary, the four major mechanisms demonstrated by ADs in reversing CRC chemotherapy resistance do not exist in isolation but form an interwoven, synergistic, and integrated system. First, the elimination of CSCs not only directly undermines the foundation for tumor regeneration and drug resistance but also indirectly suppresses the activity of pro-survival signaling pathways (such as AKT/mTOR) by reducing the expression of stemness-related factors like c-Myc and Nanog, thereby enhancing apoptosis [20,76]. Second, the reactivation of apoptosis pathways and the activation of ferroptosis not only directly kill drug-resistant cells but also promote ICD by releasing signals such as DAMPs. This further activates dendritic cells and T-cell responses, thereby reshaping the tumor immune microenvironment [40,53,54]. Furthermore, the inhibition of pro-survival signaling pathways such as PI3K/AKT and STAT3 not only weakens the internal defenses of cancer cells but also simultaneously impacts the maintenance of CSC stemness and the expression of immune-suppressive factors, forming a multi-target regulatory network [20,23,75]. Finally, the remodeling of the immune microenvironment not only enhances the ability of immune cells to recognize and eliminate tumors but also further weakens the survival niche and apoptosis resistance of CSCs by reducing inhibitory factors such as TGF-β and IL-10 [37,75,77]. Thus, ADs establish a multi-layered, multi-targeted synergistic attack network that systematically dismantles the drug resistance defense system of CRC by integrating intrinsic cellular death mechanisms with extrinsic microenvironmental regulation.

## 3. Translational Considerations of ADs in CRC

### 3.1. Clinical Pharmacokinetics, Tolerability and Safety Considerations

From a clinical pharmacokinetic perspective, ADs are characterized by rapid disposition and relatively short systemic exposure. Artesunate is rapidly hydrolyzed in vivo to its active metabolite DHA, and both artesunate and DHA exhibit short elimination half-lives in humans, typically on the order of hours, reflecting rapid systemic clearance [78,79]. As a consequence, sustained plasma and intratumoral drug exposure may be difficult to achieve in oncology settings using conventional dosing regimens. These pharmacokinetic characteristics indicate that effective anticancer translation of ADs will likely require optimized dosing schedules and/or exposure-enhancing strategies, such as formulation optimization or combination approaches, to maintain therapeutically relevant drug concentrations over time.

With respect to safety, ADs exhibit a well-established tolerability profile based on extensive antimalarial use, with reported adverse events generally being mild and transient, including gastrointestinal discomfort, dizziness, and anorexia [80]. However, rare but clinically relevant toxicities—such as post-artesunate delayed hemolysis following parenteral administration and occasional cardiac electrophysiological abnormalities—have been reported, indicating that safety profiles derived from short-course antimalarial therapy may not be fully extrapolatable to oncology settings. Therefore, hematological and electrocardiographic monitoring should be considered when ADs are repurposed for prolonged or combination-based cancer regimens [80].

Importantly, early CRC-specific clinical evidence supports translational feasibility. A randomized, double-blind, placebo-controlled pilot study of oral artesunate (200 mg daily for 14 days) in patients undergoing curative CRC resection demonstrated good short-term tolerability and biological signals consistent with anti-proliferative activity [81]. Nevertheless, the optimal dosing strategy, exposure duration, and therapeutic window for sustained anticancer efficacy in CRCs remain to be defined through dedicated oncology trials.

### 3.2. Current Formulations and Administration Routes of ADs in CRC Studies

In CRC research, ADs have been investigated using both conventional small-molecule administration and emerging delivery systems designed to overcome key translational barriers, including poor aqueous solubility, limited bioavailability, and rapid systemic clearance. Conventional administration remains prevalent in preclinical and early clinical contexts; for example, oral artesunate has demonstrated chemo preventive potential in chemically induced colon carcinogenesis models [31] and has shown feasibility and short-term tolerability in a CRC presurgical clinical trial [81].

To improve pharmacological exposure and tumor selectivity, an expanding body of work has focused on formulation and delivery innovation. Lipid-based nano systems are among the most widely explored approaches. Transferrin-conjugated liposomes have been developed to enhance artemisinin delivery by exploiting tumor-associated iron metabolism and transferrin receptor overexpression [82]. Mannosylated liposomes enabled co-delivery of DHA with doxorubicin to drug-resistant CRC models, promoting preferential intracellular and nuclear drug accumulation and the reversal of multidrug resistance [83]. Similarly, targeted lipid nanoparticles co-encapsulating DHA and chloroquine phosphate demonstrated enhanced anti-proliferative and anti-metastatic effects through redox-responsive and ligand-assisted delivery mechanisms [84].

Beyond conventional liposomes, biomimetic membrane-coated nanoparticles have been engineered to simultaneously target tumor cells and components of the tumor microenvironment. A representative example is the artesunate/chloroquine-loaded ROS-sensitive PLGA nanoparticle cloaked with mannose-modified erythrocyte membranes, which achieved dual targeting of tumor cells and tumor-associated macrophages in orthotopic CRC models and effectively remodeled the immunosuppressive microenvironment [85].

Polymer and prodrug strategies have also been employed to achieve controlled release and microenvironment responsiveness. A pH-responsive artesunate polymer prodrug capable of self-assembling into micelles enabled sustained, pH-dependent drug release and enhanced antitumor efficacy in rodent CRC models [86]. In parallel, dual-drug nano-formulations have been developed to reduce systemic toxicity and improve intratumoral accumulation, exemplified by PEG-conjugated paclitaxel–DHA nanoparticles that restrained tumor growth at lower paclitaxel doses [87].

More recent designs integrate stimulus responsiveness with immune potentiation. pH/GSH-responsive MOF-based systems have been used for targeted DHA delivery combined with carbon monoxide release, promoting ROS-driven ferroptosis, apoptosis, and ICD, thereby enhancing antitumor immunity [88]. Likewise, pH/ROS dual-responsive autocatalytic release platforms co-delivering DHA and mitoxantrone have been reported to remodel the immunosuppressive microenvironment and potentiate anti-PD-L1 immunotherapy in murine CRC models [89]. Collectively, these studies indicate that while conventional oral or parenteral dosing remains common, formulation optimization—particularly via nanocarriers, polymer prodrugs, and biomimetic systems—represents a rapidly expanding strategy to improve pharmacological exposure, tumor targeting, and immunotherapy compatibility for AD translation [80]. Representative formulations and delivery strategies of ADs across different CRC models, mechanisms, and combination regimens are systematically summarized in Table 1.

### 3.3. Translational Perspectives of ADs-Based Combination Therapy

From a translational standpoint, accumulating evidence indicates that ADs primarily function as multi-target sensitizers rather than standalone cytotoxic agents when combined with conventional chemotherapeutics. As summarized in Table 2, ADs—including DHA, artesunate, and artemisinin—exhibit synergistic antitumor effects with fluoropyrimidines, platinum-based agents, and capecitabine across diverse CRC models. Mechanistically, these combinations converge on the attenuation of CSC-associated stemness and resistance phenotypes, enhancement of oxidative vulnerability, reactivation of apoptosis and ferroptosis pathways, and remodeling of the tumor immune microenvironment [21,24].

Notably, current evidence does not demonstrate that CSCs themselves undergo classical ICD in response to artemisinin-based therapies. Instead, ADs predominantly suppress CSC-associated signaling pathways—such as AKT/mTOR and Wnt/β-catenin—thereby weakening self-renewal capacity, survival signaling, and chemoresistance [21,24]. In contrast, ICD characterized by damage-associated molecular pattern release and immune activation has mainly been observed in bulk tumor cell populations following AD-based monotherapy or combination regimens [88,89].

This functional complementarity supports a cooperative resistance-reversal model, in which suppression of CSC-associated stemness is coupled with ICD induction and immune activation in non-CSC tumor compartments. Immune-driven remodeling of the tumor microenvironment may therefore indirectly disrupt CSC-supportive niches and contribute to overcoming multidrug resistance [84,85].

With respect to targeted delivery, current artemisinin-based strategies primarily aim to enhance tumor-restricted accumulation rather than achieve CSC-exclusive targeting. These approaches include passive targeting via the enhanced permeability and retention effect [87], ligand-mediated targeting exploiting tumor iron metabolism or macrophage-associated markers [82,85], and tumor microenvironment–responsive systems triggered by pH, reactive oxygen species, or glutathione gradients [88,89]. Whether future delivery platforms can directly and selectively target CSC populations to induce bona fide ICD remains an important question for further investigation.

## 4. Conclusions and Future Perspectives

In summary, ADs represent a promising class of multi-target agents for overcoming chemotherapy resistance in colorectal cancer. By simultaneously attenuating cancer stem cell-associated resistance phenotypes, reactivating apoptosis and ferroptosis pathways, suppressing pro-survival signaling networks, and remodeling the tumor immune microenvironment, ADs collectively establish a multidimensional and synergistic antitumor strategy against chemo resistant disease.

Despite substantial advances in preclinical research, several critical challenges must be addressed to facilitate clinical translation. Future efforts should focus on the development of advanced delivery systems to improve pharmacological exposure and tumor targeting, the identification of predictive biomarkers linked to iron metabolism, redox vulnerability and stemness, and the rational optimization of combination regimens with chemotherapy and immunotherapy. In parallel, well-designed prospective clinical trials will be essential to define optimal dosing strategies, therapeutic windows, and long-term safety profiles. With continued mechanistic refinement and translational integration, artemisinin-derived compounds hold considerable promise as components of precision therapeutic strategies for CRC.

## Figures and Tables

**Table 1:** Representative Formulations and Delivery Strategies of ADs in CRC.

No.	Compound(s)	Formulation/Delivery	CRC Model(s)	Core Mechanism(s)	
1	DHA	Small-molecule (oral)	CRC patients (pre-surgery)	Anti-proliferation; biomarker modulation	[81]
2	Artesunate	Small-molecule (oral)	DMH-induced rat CRC	β-catenin inhibition; apoptosis; anti-angiogenesis	[90]
3	DHA	Nano-liposomes	HCT116, SW480	ABCG2↓; BCL2↓; oxidative stress	[91]
4	Artemisinin	Transferrin-conjugated liposomes	HCT-8	Iron-related activation; cellular uptake↑	[82]
5	DHA + Doxorubicin	Mannosylated liposomes	HCT8/ADR xenograft	Nuclear accumulation; MDR reversal	[83]
6	DHA + Paclitaxel	PEG dual-drug nanoparticles	HT-29 xenograft	Apoptosis↑; tumor accumulation↑	[87]
7	DHA + Chloroquine	Targeted lipid nanoparticles	Orthotopic CRC	ROS amplification; anti-metastasis	[84]
8	Artesunate + Chloroquine	Biomimetic PLGA nanoparticles	Orthotopic CRC	TAM repolarization; TME remodeling	[85]
9	DHA + Mitoxantrone	pH/ROS dual-responsive polymer system	CT26, MC38	ROS feedback; immune activation	[89]
10	DHA + CO donor	MOF-based nano-herb system	CRC in vivo/in vitro	Ferroptosis; apoptosis; ICD	[88]
11	Artesunate	pH-responsive polymer prodrug	CT26 xenograft	Sustained release; pH-triggered activation	[86]
12	Artemether	Albumin nanoparticles	CT26	Cytokine modulation; tumor suppression	[92]
13	Artesunate hybrids	Small-molecule derivatives	HCT116, SW480	GPX4 inhibition; ferroptosis	[93]
14	Artemisinin hybrids	Small-molecule hybrids	HCT116, HT29	ROS generation; DNA damage	[94]
15	DHA–metal complex	Ru–DHA complex	HCT116, HT-29	Cell cycle arrest; apoptosis; immune modulation	[95]

Note: ↓ Indicates a Decrease, ↑ Indicates an Increase.

**Table 2:** ADs in Combination with Chemotherapeutic Agents for CRC.

No.	ADs	CRC Model	Combined Drug	Key Mechanisms	
1	DHA	AOM/DSS-induced CRC	Capecitabine	GSK-3β/TCF7/MMP9 inhibition	[96]
2	DHA	HCT116, SW620	Oxaliplatin	AKT/mTOR inhibition; CSC suppression	[20]
3	DHA	HCT116 TP53^−/−^	5-FU	ROS-mediated apoptosis; BCL-2/BAX modulation	[55]
4	DHA	HCT116, RKO; xenograft	Oxaliplatin	ROS–STAT3/JNK/p38; PRDX2 inhibition	[64]
5	DHA	CRC cells; xenograft	Oxaliplatin	PHB2–RCHY1–p53 axis	[97]
6	DHA	CT26	Cisplatin	PERK/eIF2α-mediated ICD	[40]
7	Artesunate	MC38	Oxaliplatin	TREM2 inhibition; TAM remodeling	[98]
8	Artesunate	CRC xenograft	5-FU	Senescence suppression; mTOR inhibition	[41]
9	Artemisinin	CRC cell lines	5-FU	PI3K/AKT inhibition	[56]

## Data Availability

All data for this study are presented in the article.

## Abbreviations

The following abbreviations are used in this manuscript:

CRC	colorectal cancer
DHA	dihydroartemisinin
ADs	artemisinin-derived compounds
CSCs	cancer stem cells
ICD	immunogenic cell death
ROS	reactive oxygen species
EMT	epithelial–mesenchymal transition
ALDH	aldehyde dehydrogenase
5-FU	5-fluorouracil
DAMPs	damage-associated molecular patterns
Tregs	regulating T cells
NK	natural killer

## References

[1] Xu A., Tan T., Yuan L., Yan W., Han Y., Lu S. (2025). SUMO1 promotes malignancy and chemoresistance in colorectal cancer: Clinical and functional evidence. Tissue Cell.

[2] Shivshankar S., Patil P.S., Deodhar K., Budukh A.M. (2025). Epidemiology of colorectal cancer: A review with special emphasis on India. Indian J. Gastroenterol..

[3] Wang L., Shannar A.A.F., Wu R., Chou P., Sarwar M.S., Kuo H., Peter R.M., Wang Y., Su X., Kong A. (2022). Butyrate Drives Metabolic Rewiring and Epigenetic Reprogramming in Human Colon Cancer Cells. Mol. Nutr. Food Res..

[4] Chen X., Yang X., Chen X., Yi C., Li Z. (2025). Current approaches to overcome the limit of TRAIL-based treatment in colorectal cancer: From fusion protein to nano-delivery platform. Int. J. Pharm..

[5] Lv M.Y., Cai D., Li C.H., Chen J., Li G., Hu C., Gai B., Lei J., Lan P., Wu X. (2023). Senescence-based colorectal cancer subtyping reveals distinct molecular characteristics and therapeutic strategies. MedComm.

[6] Bahn M.S., Ko Y.G. (2023). PROM1-mediated cell signal transduction in cancer stem cells and hepatocytes. BMB Rep..

[7] Brockmueller A., Sajeev A., Koklesova L., Samuel S.M., Kubatka P., Büsselberg D., Kunnumakkara A.B., Shakibaei M. (2024). Resveratrol as sensitizer in colorectal cancer plasticity. Cancer Metastasis Rev..

[8] Erin N., Grahovac J., Brozovic A., Efferth T. (2020). Tumor microenvironment and epithelial mesenchymal transition as targets to overcome tumor multidrug resistance. Drug Resist. Updat..

[9] Chen X., Wong Y.K., Lim T.K., Lim W.H., Lin Q., Wang J., Hua Z. (2017). Artesunate Activates the Intrinsic Apoptosis of HCT116 Cells through the Suppression of Fatty Acid Synthesis and the NF-kappaB Pathway. Molecules.

[10] Mancuso R.I., Foglio M.A., Saad S.T.O. (2021). Artemisinin-type drugs for the treatment of hematological malignancies. Cancer Chemother. Pharmacol..

[11] Dai X., Zhang X., Chen W., Chen Y., Zhang Q., Mo S., Lu J. (2021). Dihydroartemisinin: A Potential Natural Anticancer Drug. Int. J. Biol. Sci..

[12] Li Q., Weina P. (2010). Artesunate: The Best Drug in the Treatment of Severe and Complicated Malaria. Pharmaceuticals.

[13] White N.J., Chotivanich K. (2024). Artemisinin-resistant malaria. Clin. Microbiol. Rev..

[14] Huang Y., Yang Y., Liu G., Xu M. (2023). New clinical application prospects of artemisinin and its derivatives: A scoping review. Infect. Dis. Poverty.

[15] Nam W., Tak J., Ryu J.K., Jung M., Yook J.I., Kim H.J., Cha I.H. (2007). Effects of artemisinin and its derivatives on growth inhibition and apoptosis of oral cancer cells. Head Neck.

[16] Lai H., Singh N.P. (2006). Oral artemisinin prevents and delays the development of 7,12-dimethylbenz[a]anthracene (DMBA)-induced breast cancer in the rat. Cancer Lett..

[17] Riganti C., Doublier S., Viarisio D., Miraglia E., Pescarmona G., Ghigo D., Bosia A. (2009). Artemisinin induce doxorubicin resistance in human colon cancer cells via calcium-dependent activation of HIF-1alpha and P-glycoprotein overexpression. Br. J. Pharmacol..

[18] Ilamathi M., Prabu P.C., Ayyappa K.A., Sivaramakrishnan V. (2016). Artesunate obliterates experimental hepatocellular carcinoma in rats through suppression of IL-6-JAK-STAT signalling. Biomed. Pharmacother..

[19] Ilamathi M., Sivaramakrishnan V. (2017). Artesunate acts as fuel to fire in sensitizing HepG2 cells towards TRAIL mediated apoptosis via STAT3 inhibition and DR4 augmentation. Biomed. Pharmacother..

[20] Wang Y., Yang Z., Zhu W., Chen Y., He X., Li J., Han Z., Yang Y., Liu W., Zhang K. (2023). Dihydroartemisinin inhibited stem cell-like properties and enhanced oxaliplatin sensitivity of colorectal cancer via AKT/mTOR signaling. Drug Dev. Res..

[21] Kumar M.S., Yadav T.T., Khair R.R., Peters G.J., Yergeri M.C. (2019). Combination Therapies of Artemisinin and its Derivatives as a Viable Approach for Future Cancer Treatment. Curr. Pharm. Des..

[22] Lu M., Sun L., Zhou J., Zhao Y., Deng X. (2015). Dihydroartemisinin-Induced Apoptosis is Associated with Inhibition of Sarco/Endoplasmic Reticulum Calcium ATPase Activity in Colorectal Cancer. Cell Biochem. Biophys..

[23] Wang D., Zhong B., Li Y., Liu X. (2018). Dihydroartemisinin increases apoptosis of colon cancer cells through targeting Janus kinase 2/signal transducer and activator of transcription 3 signaling. Oncol. Lett..

[24] Wang C.Z., Wan C., Luo Y., Zhang C.F., Zhang Q.H., Chen L., Liu Z., Wang D.H., Lager M., Li C.H. (2022). Effects of dihydroartemisinin, a metabolite of artemisinin, on colon cancer chemoprevention and adaptive immune regulation. Mol. Biol. Rep..

[25] Aswaran H., Tsai H.C., Baylin S.B. (2014). Cancer epigenetics: Tumor heterogeneity, plasticity of stem-like states, and drug resistance. Mol. Cell.

[26] Rassouli F.B., Matin M.M., Saeinasab M. (2016). Cancer stem cells in human digestive tract malignancies. Tumour Biol..

[27] Muenzner J.K., Kunze P., Lindner P., Polaschek S., Menke K., Eckstein M., Geppert C.I., Chanvorachote P., Baeuerle T., Hartmann A. (2018). Generation and characterization of hepatocellular carcinoma cell lines with enhanced cancer stem cell potential. J. Cell Mol. Med..

[28] Lau E.Y., Ho N.P., Lee T.K. (2017). Cancer Stem Cells and Their Microenvironment: Biology and Therapeutic Implications. Stem Cells Int..

[29] Walcher L., Kistenmacher A.K., Suo H., Kitte R., Dluczek S., Strauß A., Blaudszun A.R., Yevsa T., Fricke S., Boehlert U.K. (2020). Cancer Stem Cells-Origins and Biomarkers: Perspectives for Targeted Personalized Therapies. Front. Immunol..

[30] Donnenberg V.S., Donnenberg A.D. (2005). Multiple drug resistance in cancer revisited: The cancer stem cell hypothesis. J. Clin. Pharmacol..

[31] Nunes T., Hamdan D., Leboeuf C., Bouchtaoui M.E., Gapihan G., Nguyen T.T., Meles S., Angeli E., Ratajczak P., Lu H. (2018). Targeting Cancer Stem Cells to Overcome Chemoresistance. Int. J. Mol. Sci..

[32] Du B., Shim J.S. (2016). Targeting Epithelial-Mesenchymal Transition (EMT) to Overcome Drug Resistance in Cancer. Molecules.

[33] Lee S.Y., Jeong E.K., Ju M.K., Jeon H.M., Kim M.Y., Kim C.H., Park H.G., Han S.I., Kang H.S. (2017). Induction of metastasis, cancer stem cell phenotype, and oncogenic metabolism in cancer cells by ionizing radiation. Mol. Cancer.

[34] Morrison R., Schleicher S.M., Sun Y., Niermann K.J., Kim S., Spratt D.E., Chung C.H., Lu B. (2011). Targeting the mechanisms of resistance to chemotherapy and radiotherapy with the cancer stem cell hypothesis. J. Oncol..

[35] Safa A.R. (2022). Drug and apoptosis resistance in cancer stem cells: A puzzle with many pieces. Cancer Drug Resist..

[36] Wu M.H., Sung C.J., Kung F.L., Guh J.H., Su Y., Hsu L.C. (2025). Repurposing dihydroartemisinin as a novel anticancer agent against colorectal cancer stem cells. J. Food Drug Anal..

[37] Li L.N., Zhang H.D., Yuan S.J., Yang D.X., Wang L., Sun Z.X. (2008). Differential sensitivity of colorectal cancer cell lines to artesunate is associated with expression of beta-catenin and E-cadherin. Eur. J. Pharmacol..

[38] Li L.N., Zhang H.D., Yuan S.J., Tian Z.Y., Wang L., Sun Z.X. (2007). Artesunate attenuates the growth of human colorectal carcinoma and inhibits hyperactive Wnt/beta-catenin pathway. Int. J. Cancer.

[39] Hamoya T., Fujii G., Iizumi Y., Narita T., Komiya M., Matsuzawa Y., Miki K., Kondo T., Kishimoto S., Watanabe K. (2021). Artesunate inhibits intestinal tumorigenesis through inhibiting wnt signaling. Carcinogenesis.

[40] Li Y., Ma P., Li J., Wu F., Guo M., Zhou E., Song S., Wang S., Zhang S., Jin Y. (2024). Dihydroartemisinin restores the immunogenicity and enhances the anticancer immunosurveillance of cisplatin by activating the PERK/eIF2alpha pathway. Cell Biosci..

[41] Xia J., Dai Q.L., He S., Jia H.J., Liu X.G., Hua H., Zhou M., Wang X. (2023). Artesunate alleviates 5-fluorouracil-induced intestinal damage by suppressing cellular senescence and enhances its antitumor activity. Discov. Oncol..

[42] Dekker E., Tanis P.J., Vleugels J., Kasi P.M., Wallace M.B. (2019). Colorectal cancer. Lancet.

[43] Chen T., Yang I., Irby R., Shain K.H., Wang H.G., Quackenbush J., Coppola D., Cheng J.Q., Yeatman T.J. (2003). Regulation of caspase expression and apoptosis by adenomatous polyposis coli. Cancer Res..

[44] Green R.A., Kaplan K.B. (2003). Chromosome instability in colorectal tumor cells is associated with defects in microtubule plus-end attachments caused by a dominant mutation in APC. J. Cell Biol..

[45] Hu T., Wang L., Zhang L., Lu L., Shen J., Chan R.L.Y., Li M., Wu W.K.K., To K.K.W., Cho C.H. (2015). Sensitivity of apoptosis-resistant colon cancer cells to tanshinones is mediated by autophagic cell death and p53-independent cytotoxicity. Phytomedicine.

[46] Hu T., Li Z., Gao C.Y., Cho C.H. (2016). Mechanisms of drug resistance in colon cancer and its therapeutic strategies. World J. Gastroenterol..

[47] Mita A.C., Mita M.M., Nawrocki S.T., Giles F.J. (2008). Survivin: Key regulator of mitosis and apoptosis and novel target for cancer therapeutics. Clin. Cancer Res..

[48] Cheung C.H.A., Huang C.C., Tsai F.Y., Lee J.Y.C., Cheng S.M., Chang Y.C., Huang Y.C., Chen S.H., Chang J.Y. (2013). Survivin-biology and potential as a therapeutic target in oncology. OncoTargets Ther..

[49] Lu M., Sun L., Zhou J., Yang J. (2014). Dihydroartemisinin induces apoptosis in colorectal cancer cells through the mitochondria-dependent pathway. Tumour Biol..

[50] Lu J.J., Chen S.M., Zhang X.W., Ding J., Meng L.H. (2011). The anti-cancer activity of dihydroartemisinin is associated with induction of iron-dependent endoplasmic reticulum stress in colorectal carcinoma HCT116 cells. Investig. New Drugs.

[51] Dixon S.J., Lemberg K.M., Lamprecht M.R., Skouta R., Zaitsev E.M., Gleason C.E., Patel D.N., Bauer A.J., Cantley A.M., Yang W.S. (2012). Ferroptosis: An iron-dependent form of nonapoptotic cell death. Cell.

[52] Lei G., Mao C., Yan Y., Zhuang L., Gan B. (2021). Ferroptosis, radiotherapy, and combination therapeutic strategies. Protein Cell.

[53] Wei X., Wu J., Zhu C., Yu M., Niu N., Lv H., Chen G. (2025). Dihydroartemisinin Suppresses LOXL2-Mediated Glycerophospholipid Metabolic Reprogramming to Induce Cuproptosis in Colorectal Cancer Cells. J. Biochem. Mol. Toxicol..

[54] Bader S., Wilmers J., Pelzer M., Jendrossek V., Rudner J. (2021). Activation of anti-oxidant Keap1/Nrf2 pathway modulates efficacy of dihydroartemisinin-based monotherapy and combinatory therapy with ionizing radiation. Free Radic. Biol. Med..

[55] Yao Z., Bhandari A., Wang Y., Pan Y., Yang F., Chen R., Xia E., Wang Q. (2018). Dihydroartemisinin potentiates antitumor activity of 5-fluorouracil against a resistant colorectal cancer cell line. Biochem. Biophys. Res. Commun..

[56] Wang Y., Chen Y., Liu L., He D., Li H. (2020). Effects of Artemisinin Combined with 5-Fluorouracil on Colon Cancer Cell Proliferation, Migration, and Drug Sensitivity via PI3K/AKT Signaling. J. Biomater. Tissue Eng..

[57] Almaimani R.A., Aslam A., Ahmad J., El-Readi M.Z., El-Boshy M.Z., Abdelghany A.H., Idris S., Alhadrami M., Althubiti M., Almasmoum H.A. (2022). In Vivo and In Vitro Enhanced Tumoricidal Effects of Metformin, Active Vitamin D (3), and 5-Fluorouracil Triple Therapy against Colon Cancer by Modulating the PI3K/Akt/PTEN/mTOR Network. Cancers.

[58] Xiong M., Zhuang K., Luo Y., Lai Q., Luo X., Fang Y., Zhang Y., Li A., Liu S. (2019). KIF20A promotes cellular malignant behavior and enhances resistance to chemotherapy in colorectal cancer through regulation of the JAK/STAT3 signaling pathway. Aging.

[59] Wang X., Lai Q., He J., Li Q., Ding J., Lan Z., Gu C., Yan Q., Fang Y., Zhao X. (2019). LncRNA SNHG6 promotes proliferation, invasion and migration in colorectal cancer cells by activating TGF-beta/Smad signaling pathway via targeting UPF1 and inducing EMT via regulation of ZEB1. Int. J. Med. Sci..

[60] Bhattacharya S., Ray R.M., Johnson L. (2005). STAT3-mediated transcription of Bcl-2, Mcl-1 and c-IAP2 prevents apoptosis in polyamine-depleted cells. Biochem. J..

[61] Yue Y., Zhang Q., Wang X., Sun Z. (2023). STAT3 regulates 5-Fu resistance in human colorectal cancer cells by promoting Mcl-1-dependent cytoprotective autophagy. Cancer Sci..

[62] Gui Y., Qian X., Ding Y., Chen Q., Ye F., Ye Y., Hou Y., Yu J., Zhao L. (2024). c-Fos regulated by TMPO/ERK axis promotes 5-FU resistance via inducing NANOG transcription in colon cancer. Cell Death Dis..

[63] Shen X., Shi C., Lei M., Zhou R., Liu S., Su C. (2024). Dihydroartemisinin, an artemisinin derivative, reverses oxaliplatin resistance in human colorectal cancer cells by regulating the SIRT3/PI3K/AKT signalling pathway. Investig. Clin..

[64] Yu Y., Chen D., Wu T., Lin H., Ni L., Sui H., Xiao S., Wang C., Jiang S., Pan H. (2022). Dihydroartemisinin enhances the anti-tumor activity of oxaliplatin in colorectal cancer cells by altering PRDX2-reactive oxygen species-mediated multiple signaling pathways. Phytomedicine.

[65] Ni Q., Li M., Yu S. (2022). Research Progress of Epithelial-mesenchymal Transition Treatment and Drug Resistance in Colorectal Cancer. Technol. Cancer Res. Treat..

[66] Zhang Q., Ding J., Wang Y., He L., Xue F. (2022). Tumor microenvironment manipulates chemoresistance in ovarian cancer (Review). Oncol. Rep..

[67] Gallo G., Vescio G., Paola G.D., Sammarco G. (2021). Therapeutic Targets and Tumor Microenvironment in Colorectal Cancer. J. Clin. Med..

[68] Waldner M.J., Neurath M.F. (2023). TGFbeta and the Tumor Microenvironment in Colorectal Cancer. Cells.

[69] Li S., Xie M., Du Y., Tan Z. (2025). Targeting cytokine and chemokine signaling pathways for enhancing chemo-sensitivity in colorectal cancer. Cell Commun. Signal..

[70] Shen Z., Li Z., Liu Y., Li Y., Feng X., Zhan Y., Lin M., Fang C., Fang Y., Deng H. (2022). GLUT5-KHK axis-mediated fructose metabolism drives proliferation and chemotherapy resistance of colorectal cancer. Cancer Lett..

[71] Li J., Chen D., Shen M. (2022). Tumor Microenvironment Shapes Colorectal Cancer Progression, Metastasis, and Treatment Responses. Front. Med..

[72] Chen T.W., Hung W.Z., Chiang S.F., Chen W., Ke T.W., Liang J.A., Huang C.Y., Yang P.C., Huang K., Chao K. (2022). Dual inhibition of TGFbeta signaling and CSF1/CSF1R reprograms tumor-infiltrating macrophages and improves response to chemotherapy via suppressing PD-L1. Cancer Lett..

[73] Kousar K., Ahmad T., Abduh M.S., Kanwal B., Shah S.S., Naseer F., Anjum S. (2022). miRNAs in Regulation of Tumor Microenvironment, Chemotherapy Resistance, Immunotherapy Modulation and miRNA Therapeutics in Cancer. Int. J. Mol. Sci..

[74] Wang Y., Liang H., Zheng J. (2022). Exosomal microRNAs mediating crosstalk between cancer cells and cancer-associated fibroblasts in the tumor microenvironment. Pathol. Res. Pract..

[75] Cui C., Feng H., Shi X., Wang Y., Feng Z., Liu J., Han Z., Fu J., Fu Z., Tong H. (2015). Artesunate down-regulates immunosuppression from colorectal cancer Colon26 and RKO cells in vitro by decreasing transforming growth factor beta1 and interleukin-10. Int. Immunopharmacol..

[76] Hu X., Fatima S., Chen M., Huang T., Chen Y.W., Gong R., Wong H., Yu R., Song L., Kwan H.Y. (2021). Dihydroartemisinin is potential therapeutics for treating late-stage CRC by targeting the elevated c-Myc level. Cell Death Dis..

[77] Bader S., Wilmers J., Ontikatze T., Ritter V., Endrossek V., Rudner J. (2021). Loss of pro-apoptotic Bax and Bak increases resistance to dihydroartemisinin-mediated cytotoxicity in normoxia but not in hypoxia in HCT116 colorectal cancer cells. Free Radic. Biol. Med..

[78] Watson D.J., Laing L., Gibhard L., Wong H.N., Haynes R.K., Wiesner L. (2021). Toward New Transmission-Blocking Combination Therapies: Pharmacokinetics of 10-Amino-Artemisinins and 11-Aza-Artemisinin and Comparison with Dihydroartemisinin and Artemether. Antimicrob. Agents Chemother..

[79] Morris C.A., Duparc S., Isabelle B.F., Jung D., Shin C.S., Fleckenstein L. (2011). Review of the clinical pharmacokinetics of artesunate and its active metabolite dihydroartemisinin following intravenous, intramuscular, oral or rectal administration. Malar. J..

[80] Li J., Zheng J., Cui Y., Liu Y., Fan H., Wang X., Liu H., Li X., Yu G., Luo Z. (2025). The Role of Artesunate in Cancer Management: Mechanisms of Biomedical Effects and Toxicology. Am. J. Chin. Med..

[81] Krishna S., Ganapathi S., Ster I.C., Saeed M.E.M., Cowan M., Finlayson C., Kovacsevics H., Jansen H., Kremsner P.G., Efferth T. (2014). A Randomised, Double Blind, Placebo-Controlled Pilot Study of Oral Artesunate Therapy for Colorectal Cancer. EBioMedicine.

[82] Leto I., Coronnello M., Righeschi C., Bergonzi M.C., Mini E., Bilia A.R. (2016). Enhanced Efficacy of Artemisinin Loaded in Transferrin-Conjugated Liposomes versus Stealth Liposomes against HCT-8 Colon Cancer Cells. ChemMedChem.

[83] Kang X.J., Wang H.Y., Peng H.G., Chen B.F., Zhang W.Y., Wu A.H., Xu Q., Huang Y.Z. (2017). Codelivery of dihydroartemisinin and doxorubicin in mannosylated liposomes for drug-resistant colon cancer therapy. Acta Pharmacol. Sin..

[84] Peng J., Wang Q., Zhou J., Zhao S., Di P., Chen Y., Tao L., Du Q., Shen X., Chen Y. (2021). Targeted Lipid Nanoparticles Encapsulating Dihydroartemisinin and Chloroquine Phosphate for Suppressing the Proliferation and Liver Metastasis of Colorectal Cancer. Front. Pharmacol..

[85] Peng J., Zhou J., Sun R., Chen Y., Pan D., Wang Q., Chen Y., Gong Z., Du Q. (2023). Dual-targeting of artesunate and chloroquine to tumor cells and tumor-associated macrophages by a biomimetic PLGA nanoparticle for colorectal cancer treatment. Int. J. Biol. Macromol..

[86] Hao D.L., Xie R., De G.J., Yi H., Zang C., Yang M.Y., Liu L., Ma H., Cai W.Y., Zhao Q.H. (2020). pH-Responsive Artesunate Polymer Prodrugs with Enhanced Ablation Effect on Rodent Xenograft Colon Cancer. Int. J. Nanomed..

[87] Phung C.D., Le T.G., Nguyen V.H., Vu T.T., Nguyen H.Q., Kim J.O., Yong C.S., Nguyen C.N. (2020). PEGylated-Paclitaxel and Dihydroartemisinin Nanoparticles for Simultaneously Delivering Paclitaxel and Dihydroartemisinin to Colorectal Cancer. Pharm. Res..

[88] Yang C., Ming H., Li B., Liu S., Chen L., Zhang T., Gao Y., He T., Huang C., Du Z. (2024). A pH and glutathione-responsive carbon monoxide-driven nano-herb delivery system for enhanced immunotherapy in colorectal cancer. J. Control. Release Off. J. Control. Release Soc..

[89] Su Q., Wang Z., Li P., Wei X., Xiao J., Duan X. (2024). pH and ROS Dual-Responsive Autocatalytic Release System Potentiates Immunotherapy of Colorectal Cancer. Adv. Healthc. Mater..

[90] Verma S., Das P., Kumar V.L. (2017). Chemoprevention by artesunate in a preclinical model of colorectal cancer involves down regulation of β-catenin, suppression of angiogenesis, cellular proliferation and induction of apoptosis. Chem.-Biol. Interact..

[91] Cao J.F., Hang K., Tan C., Wu Z., Guo Z., Xia Q., Liao D., Xia Z., Men J., Li K. (2026). Targeted inhibition of colorectal cancer by nano-dihydroartemisinin liposomes: Insights into the disruption of ABCG2 and BCL2 leading to induction of oxidative stress and metabolic disorder. Biomater. Adv..

[92] Pirali-Hamedani Z., Abbasi A., Hassan Z.M. (2022). Synthesis of Artemether-Loaded Albumin Nanoparticles and Measurement of Their Anti-Cancer Effects. Biomedicines.

[93] Ren M., Liang S., Lin S., Huang R., Chen Y., Zhang Y., Xu Y. (2024). Design, synthesis and biological evaluation of artesunate-Se derivatives as anticancer agents by inducing GPX4-mediated ferroptosis. Bioorganic Chem..

[94] Fröhlich T., Ndreshkjana B., Muenzner J.K., Reiter C., Hofmeister E., Mederer S., Fatfat M., El-Baba C., Gali-Muhtasib H., Schneider-Stock R. (2017). Synthesis of Novel Hybrids of Thymoquinone and Artemisinin with High Activity and Selectivity Against Colon Cancer. ChemMedChem.

[95] Wang C.Z., Wan C., Li C.H., Liang G.G., Luo Y., Zhang C., Zhang Q.H., Ma Q., Wang A.H., Lager M. (2024). Ruthenium-dihydroartemisinin complex: A promising new compound for colon cancer prevention via G1 cell cycle arrest, apoptotic induction, and adaptive immune regulation. Cancer Chemother. Pharmacol..

[96] Dai X., Chen W., Qiao Y., Chen X., Chen Y., Zhang K., Zhang Q., Duan X., Li X., Zhao J. (2024). Dihydroartemisinin inhibits the development of colorectal cancer by GSK-3β/TCF7/MMP9 pathway and synergies with capecitabine. Cancer Lett..

[97] Wang X., Zheng Y., Chai Z., Li J., Zhu C., Peng Y., Qiu J., Xu J., Liu C. (2023). Dihydroartemisinin synergistically enhances the cytotoxic effects of oxaliplatin in colon cancer by targeting the PHB2-RCHY1 mediated signaling pathway. Mol. Carcinog..

[98] Yang T., Zhang S., Yuan H., Wang Y., Cai L., Chen H., Wang X., Song D., Wang X., Guo Z. (2023). Platinum-Based TREM2 Inhibitor Suppresses Tumors by Remodeling the Immunosuppressive Microenvironment. Angew. Chem. Int. Ed. Engl..

